# Children with Duchenne muscular dystrophy display specific kinematic strategies during obstacle-crossing

**DOI:** 10.1038/s41598-023-44270-9

**Published:** 2023-10-10

**Authors:** Kuan-Wen Wu, Cheng-Hao Yu, Tse-Hua Huang, Shiuan-Huei Lu, Yu-Lin Tsai, Ting-Ming Wang, Tung-Wu Lu

**Affiliations:** 1 Department of Orthopaedic Surgery, School of Medicine, National Taiwan University, Taipei, Taiwan, ROC; 2 Department of Orthopaedic Surgery, National Taiwan University Hospital, Taipei, Taiwan, ROC; 3 Department of Biomedical Engineering, National Taiwan University, Taipei, Taiwan, ROC; 4 Department of Mechanical Engineering, National Taiwan University, Taipei, Taiwan, ROC

**Keywords:** Risk factors, Biomedical engineering, Disability, Motor control

## Abstract

Duchenne muscular dystrophy (DMD) is a genetic disease characterized by progressive muscle weakness with increased neuromechanical challenge and fall risks, especially during obstructed locomotion. This study aimed to identify the kinematic strategies for obstacle-crossing in DMD via synthesizing the changes in the joint kinematics and associated end-point control. Fourteen boys with DMD (age: 9.0 ± 2.5 years) and fourteen typically developed controls (age: 9.0 ± 2.8 years) each crossed obstacles of three different heights (10%, 20% and 30% of leg length) while the angular motions of the trunk-pelvis-leg apparatus and foot-obstacle clearances were measured. Two-way analyses of variance were used to analyze group and obstacle height effects. Compared to the controls, the DMD group crossed obstacles with significantly increased step width, but decreased crossing speed, crossing step length, trailing toe-obstacle clearance and leading heel-obstacle horizontal distance (*p* < 0.05). When the leading toe was above the obstacle, the patients showed significantly increased pelvic hiking, pelvic and trunk anterior tilt and ankle plantarflexion, but decreased hip flexion in both limbs (*p* < 0.05). Similar kinematic changes were found during trailing-limb crossing, except for an additional increase in swing-hip abduction and decrease in contralateral trunk side-bending and stance-knee flexion. Patients with DMD crossed obstacles via a specific kinematic strategy with altered end-point control, predisposing them to a greater risk of tripping during trailing-limb crossing. These results suggest that crossing kinematics in DMD should be monitored—especially in the proximal segments of the pelvis-leg apparatus—that may lead to an increased risk of falling.

## Introduction

Duchenne Muscular Dystrophy (DMD) is a progressive, genetically defective neuromuscular disease caused by a lack of dystrophin protein, making muscle fibers fragile and prone to death^1^. One in every 3,500 births worldwide suffers from DMD^1–4^. The muscular dystrophy starts primarily in the proximal lower limbs, especially in the hip muscles, affecting the patient's daily locomotion^5^. The progressive muscle degeneration and replacement with fibrotic and fatty tissue lead to muscle tear and muscle atrophy, contributing to reduced strength and dysfunctionality^1^. Muscular weakness or dystrophy in the lower limbs contributes to early fatigue, unstable and altered locomotion patterns, as well as abnormal postural control and balance disturbances during gait, leading to an increased risk of falling^6–9^. During daily locomotion, obstacles are a major environmental hazard leading to falls and serious injuries in those with compromised neuromusculoskeletal functions^10^. Obstacle-negotiation requires high motor execution and balance control ability. It is thus a task that exposes children with DMD to an increased risk of falling^6–9^. Therefore, identifying kinematic changes during obstacle-crossing will be useful for developing strategies to reduce the risk of falling and the associated injuries in children with DMD.

Deficits in muscle strength and muscular control have been shown to limit walking capabilities in patients with DMD. Proximal muscular weakness contributes to difficulties in generating required joint moments for propulsion and maintaining stability during walking^11^. As a compensatory mechanism, patients with DMD may exhibit a bilateral Trendelenburg gait characterized by lateral trunk side-bending in combination with a contralateral pelvic drop during single-limb support, owing to the atrophy of the gluteal musculature^12–15^. Gait studies on patients with DMD have shown reduced speed and stride length but greater stride width and increased peak extension and ranges of motion at the knee when compared to typically developed controls^8,16^. Owing to the weakness of the quadriceps femoris, the knee joint is excessively extended in order to maintain stability during stance phase^8^. Weakness of the hip and proximal thigh muscles leads to reduced adduction and flexion at the hip joint during swing, and thus reduces the floor clearance of the foot, increasing the risk of tripping^16,17^. Patients with DMD would be expected to have more difficulties than their typically developed peers in dealing with the neuromechanical challenges during obstacle-crossing. It is known that the risk of falling increases linearly with the number of risk factors^18–22^. Thus, the cumulative effects of DMD and obstacle-crossing may predispose a child with DMD to an increased risk of falling. Identifying the changes in joint angular motions, toe-obstacle clearances and foot-obstacle distances will be helpful for a better understanding of the kinematic deviations caused and the compensatory strategies required or developed to overcome the challenges during obstacle-crossing in patients with DMD. However, to the best knowledge of the authors, no studies on the kinematics of the trunk and the pelvis-leg apparatus in DMD during obstacle-crossing have been reported in the literature.

During obstacle-crossing, the motions of the individual joints are controlled to maintain dynamic balance while allowing the swing-limb to cross the obstacle with sufficient foot-obstacle clearance. Any alterations of the joint kinematics resulting from injury or pathology of the neuromusculoskeletal system will affect the inter-joint coordination and end-point control for successful obstacle-crossing. Such inter-joint and joint-to-end-point kinematic relationships can vary among subject groups and motor tasks, reflecting the neuromusculoskeletal control of the person. However, such kinematic coordination is difficult to identify by examining the changes in individual joints and the end-points. A multi-link system approach has been successfully used in synthesizing the kinematic changes of individual joints and end-points to identify the kinematic strategies of obstacle-crossing in various populations^23–25^. Considering the human pelvis-leg apparatus as a multi-link system, a change in the angle of a joint will lead to angular changes at other joints, which together determine the end-point position of the swing limb. Analyses of the kinematic changes in the joints and end-points during obstacle-crossing using the multi-link approach enable the kinematic control strategies adopted during obstacle-crossing and the risk factors for falling to be identified^26–28^. Using such an approach, knowledge of the kinematic and associated end-point control strategies adopted by patients with DMD during obstacle-crossing could be obtained.

The purposes of the current study were to quantify the kinematic changes of the trunk, and the individual joints and end-points of the pelvis-leg apparatus in children with DMD during obstacle-crossing in comparison with their typically developed peers, and to identify the kinematic strategy adopted by the DMD group. It was hypothesized that children with DMD would adopt a specific compensatory kinematic strategy with altered joint kinematics and end-point positions for obstacle-crossing, and that these strategies would be affected by obstacle height.

## Materials and methods

### Subjects

In the current observational cross-sectional study, participants with DMD aged over 6–14 years were recruited from the university hospital (DMD Group), and typically developed healthy peers, matched in age, body height and body mass, were recruited from community schools (Control group), between December 2019 and December 2021. The inclusion criteria for the DMD group were: (1) diagnosed with DMD by a senior pediatrician via immunohistochemistry, muscle biopsy or mutation of the dystrophin gene; (2) no history of lower-limb surgery; (3) no central nervous system lesion or other severe neuromusculoskeletal disorders^29^. A participant would be excluded if he could not walk and cross obstacles independently or communicate to complete the clinician’s interview. All participants underwent manual muscle testing (MMT) of the gluteus maximus, rectus femoris, tibialis anterior, biceps femoris and gastrocnemius by an experienced therapist (SHL) according to the Medical Research Council scale (MRC scale)^30,31^. For the purpose of subsequent data analysis, a grade marked “ − ” was considered to be the grade minus 0.33, and a grade marked “ + ” was considered to be the grade plus 0.33 (for example: 3 +  = 3.33)^6,8^. The experiments and procedures in the current study conformed to the Ethical Principles for Medical Research Involving Human Subjects (World Medical Association Declaration of Helsinki). Written informed assents were obtained from the participants and written informed consents were obtained from their parents or guardians as approved by the Institutional Review Board (Permit number: 201912113RINB).

### Gait experiments

Each subject walked at their preferred speed on a 10-m walkway and crossed a tube-like obstacle placed horizontally across a height-adjustable frame^32^. Two infrared retroreflective markers on either end of the tube defined the position and height of the obstacle. Thirty-nine infrared retroreflective markers attached to specific anatomical landmarks tracked the motions of all the body segments, namely anterior superior iliac spines, posterior superior iliac spines, greater trochanters, mid-thighs, medial and lateral epicondyles, heads of fibulae, tibial tuberosities, medial and lateral malleoli, navicular tuberosities, fifth metatarsal bases, big toes and heels, and mandibular condylar processes, acromion processes, spinous processes of the seventh cervical vertebra (C7), medial and lateral humeral epicondyles, and ulnar styloids^27,28,33^. An eight-camera motion analysis system (Vicon MX T-40, OMG, U.K.) measured the three-dimensional marker trajectories at 200 Hz, while the ground reaction forces (GRF) were measured at 2000 Hz using three forceplates (OR6-7, AMTI, U.S.A.) placed on either side of the obstacle in the middle of the walkway. Each subject walked and crossed obstacles of three heights (*i.e.*, 10%, 20%, and 30% of the subject’s leg length) in a random order decided by a random number table with a counterbalanced measures design. For each obstacle height, data for three complete crossing cycles with each of the right and left lower limbs leading were obtained for each subject.

### Data analysis

Utilizing the measured marker data, each body segment was embedded with a Cartesian coordinate system. The positive x-axis, y-axis and z-axis were pointed anteriorly, superiorly and to the right, respectively^34^. When describing the kinematics of the body segments and joints, the leading limb was designated as the reference limb, so the term “ipsilateral” refers to the leading limb side and “contralateral” refers to the trailing limb side. The pelvic and trunk motions were described relative to the laboratory coordinate system and the motions of the trunk were also described relative to the pelvic system (here referred to as trunk/pelvis). The Cardanic rotation sequence of y-x-z was used to describe these rotational movements. For the motions in the frontal plane, pelvic hiking indicates that the ipsilateral hip has risen above the contralateral hip^28,35–38^, the ipsilateral side-bending of the trunk and trunk/pelvis is defined as the trunk rotational deviation to the ipsilateral side in relation to the laboratory and pelvic coordinate system respectively^35,39,40^. Using a z-x-y Euler rotation sequence, the lower-limb joint angles were also extracted from the transformation matrices between the distal segment relative to the proximal^41^. To reduce the effects of soft tissue artefacts of the skin markers attached to the pelvis-leg apparatus, a global optimization method with joint constraints and an error compensation mechanism was applied to the marker data^42^. The foremost mentioned pelvic and trunk orientations, trunk/pelvis motions and joint angles were calculated for the crossing cycle, defined as the duration from leading toe-off immediately before crossing to leading toe-off immediately after crossing. Their values when the leading and trailing toes were above the obstacle, referred to as crossing angles, were extracted for the subsequent statistical analysis^43^.

Temporospatial and end-point parameters were also obtained. For temporospatial gait parameters, the crossing speed was calculated as the distance travelled by mid-point between the anterior superior iliac spines in the walking direction divided by the time spent during the crossing cycle. The crossing step length was calculated as the distance between the leading and trailing heel markers at respective heel-strike points along the walking direction, and the perpendicular distance was deemed as the crossing step width. For end-point parameters, the vertical distance from the toe marker to the obstacle at the crossing moment was calculated as the toe-obstacle clearance. Similarly, heel-obstacle clearance was calculated as the swing-heel marker was directly above the obstacle. The horizontal distance between the obstacle and the trailing stance limb’s toe marker before stepping over the obstacle defined the trailing toe-obstacle horizontal distance, whereas the distance between the obstacle and the leading limb’s heel marker at heel-strike after crossing defined the leading heel-obstacle horizontal distance^39^.

### Statistical analysis

Statistical analyses were performed on the temporospatial and end-point parameters, and the angles of the trunk, pelvis, trunk/pelvis, and lower-limb joints in both the frontal and sagittal planes when the leading and trailing toes were positioned above the obstacle. Data of each calculated variable from both sides were first averaged before the means were calculated for each trial. The normality of all the calculated variables was tested using the Shapiro–Wilk test. A two-way mixed-design analysis of variance (ANOVA) with one between-subject main factor (group) and one within-subject main factor (obstacle height) was conducted for all variables. In the absence of significant interactions, main effects were reported. A post hoc analysis was further applied when a significant obstacle height effect was found, using a polynomial test to determine the linear trend. A *p* value of ≤ 0.05 was considered statistically significant for all tests. All statistical analyses were conducted using SPSS statistics (Version 20, SPSS Inc., Chicago, IL, U.S.A.). The relationship between significant kinematic changes at end-points and individual joints was synthesized using the multi-link system approach^24,39,44^ to further differentiate the kinematic strategies in performing obstacle-crossing between DMD and the Control group.

### Sample size

An a priori power analysis using G*POWER^45^ based on data from previous studies^35,39^ determined that a projected sample size of four subjects for each group would be needed for a power of 0.8 and a large effect size (Cohen’s d = 0.9) at a significance level of 0.05.

## Results

The current study recruited fourteen boys with Duchenne muscular dystrophy (DMD group; age: 9.0 ± 2.5 years, height: 128.0 ± 15.8 cm, mass: 35.5 ± 14.9 kg) and fourteen typically developed healthy boys (Control group; age: 9.0 ± 2.8 years, height: 122.0 ± 22.7 cm, body mass: 31.0 ± 11.5 kg), matched in age, body height and body mass (Table 1). The results of MMT showed significantly reduced strength in the gluteus maximus, rectus femoris, and tibialis anterior muscles in the DMD Group as compared to the Control while the biceps femoris and gastrocnemius muscles were of similar strength in both groups (Table 1).

**Table 1:** Means (standard deviations) of the demographic characteristic and manual muscle testing (MMT) scores of subjects in the Duchenne muscular dystrophy group (DMD, n = 14) and typically developed controls (Control, n = 14).

	DMD	Control	*p*-value
Age (years)	9.0 (2.48)	9.0 (2.75)	0.999
Body height (cm)	128.0 (15.75)	122.0 (22.72)	0.423
Body mass (kg)	33.51 (14.90)	31.0 (11.53)	0.619
Manual muscle testing scores			
Gluteus Maximus	3.59 (0.71)	5.00 (0.00)	< 0.001*
Rectus Femoris	3.90 (0.61)	5.00 (0.00)	< 0.001*
Tibialis Anterior	3.78 (0.62)	5.00 (0.00)	< 0.001*
Biceps Femoris	4.71 (0.10)	5.00 (0.00)	0.104
Gastrocnemius	4.78 (0.18)	5.00 (0.00)	0.082

*p < 0.05.

**Figure 1:**
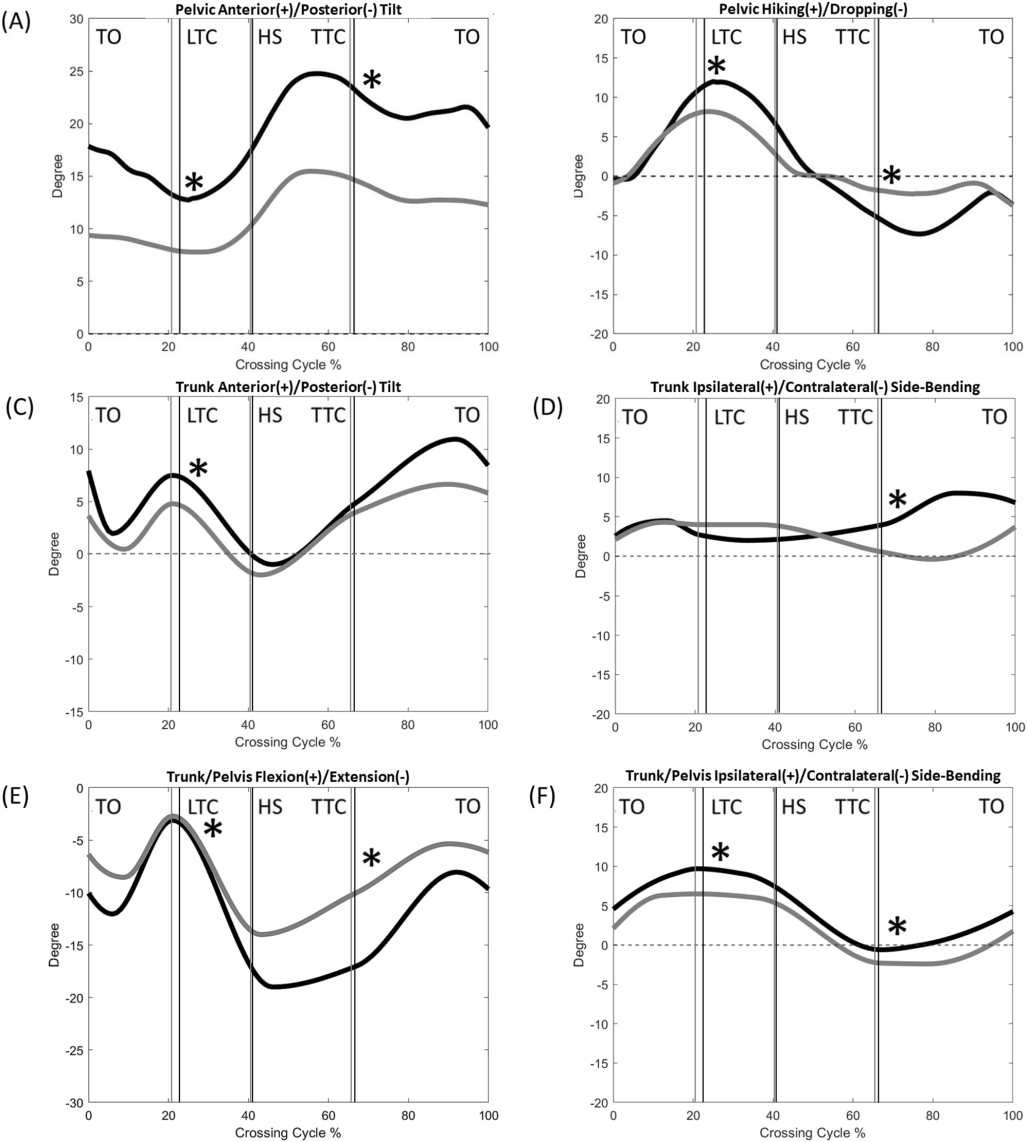
The mean curves of the angles of the pelvis and trunk segments, as well as trunk/pelvis in the DMD (black) and Control (grey) groups when crossing obstacles of 20% of leg length. (TO: leading toe-off; LTC: leading toe above the obstacle; HS: leading heel-strike; TTC: trailing toe above the obstacle; *: significant main group effect, *p* < 0.05).

**Figure 2:**
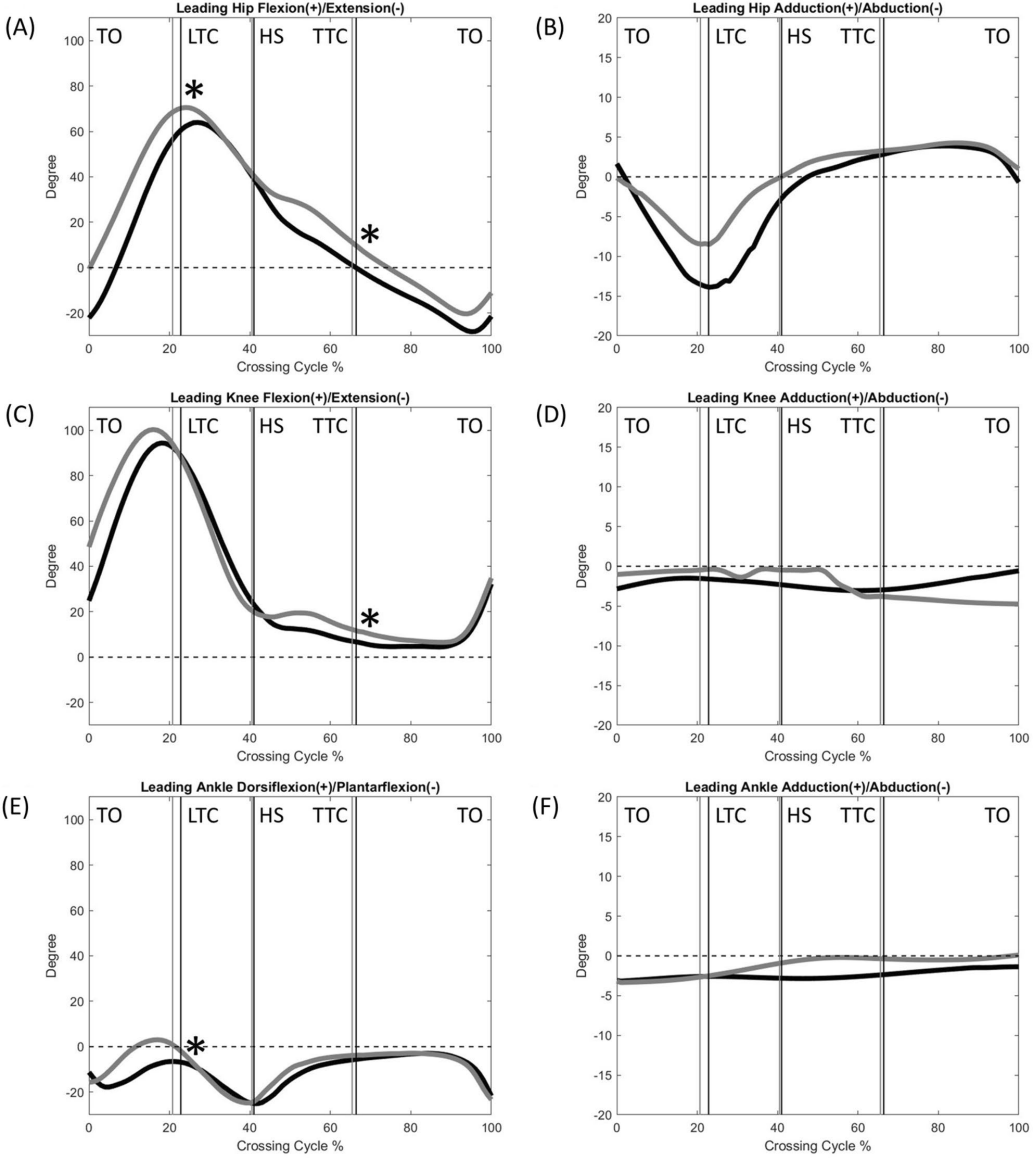
The mean curves of the angles of hip, knee and ankle joints of the leading limb in the DMD (black) and Control (grey) groups when crossing obstacles of 20% of leg length. (TO: leading toe-off; LTC: leading toe above the obstacle; HS: leading heel-strike; TTC: trailing toe above the obstacle; *: significant main group effect, *p* < 0.05).

**Figure 3:**
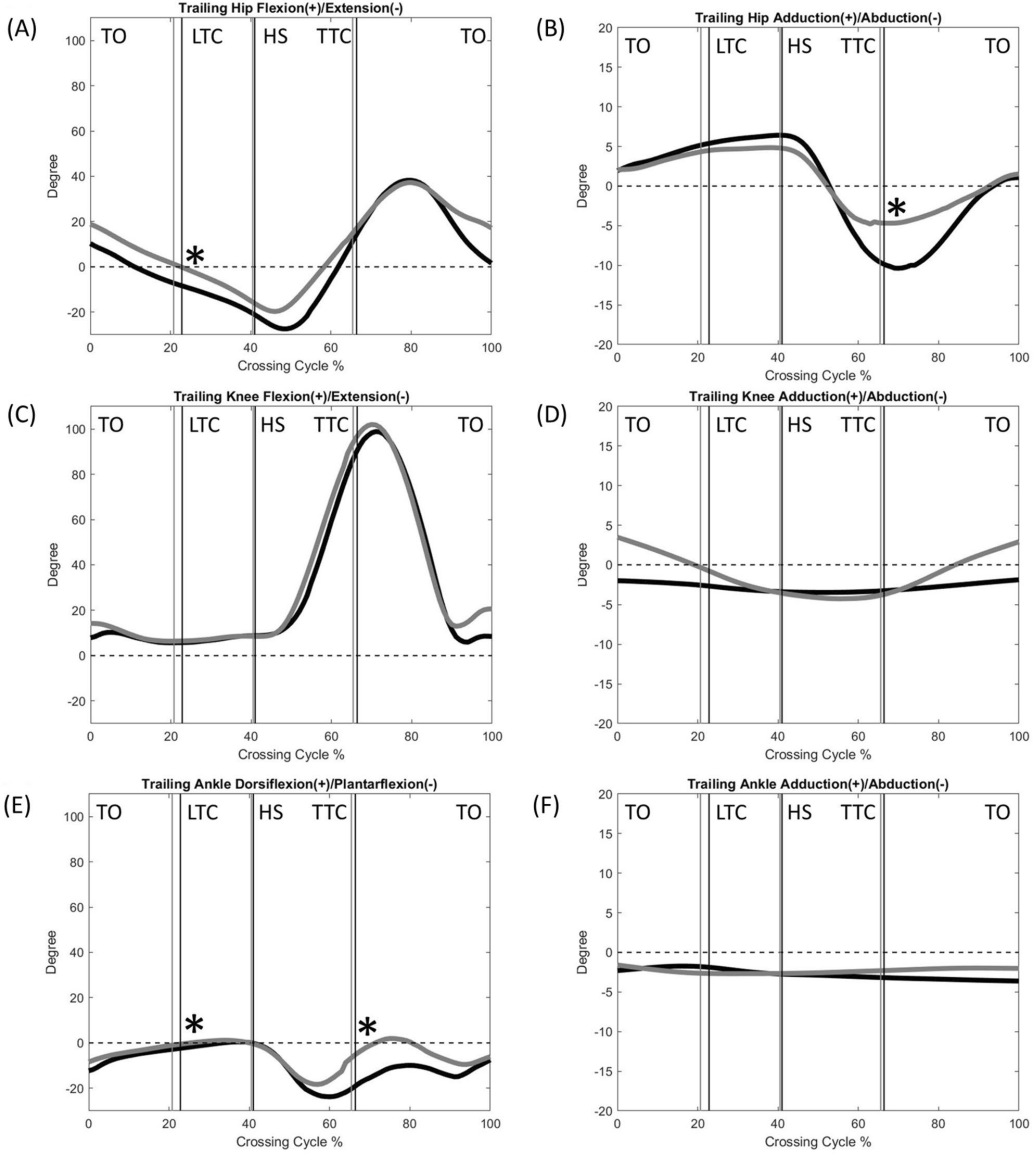
The mean curves of the angles of hip, knee and ankle joints of the trailing limb in the DMD (black) and Control (grey) groups when crossing obstacles of 20% of leg length. (TO: leading toe-off; LTC: leading toe above the obstacle; HS: leading heel-strike; TTC: trailing toe above the obstacle; *: significant main group effect, *p* < 0.05).

**Figure 4:**
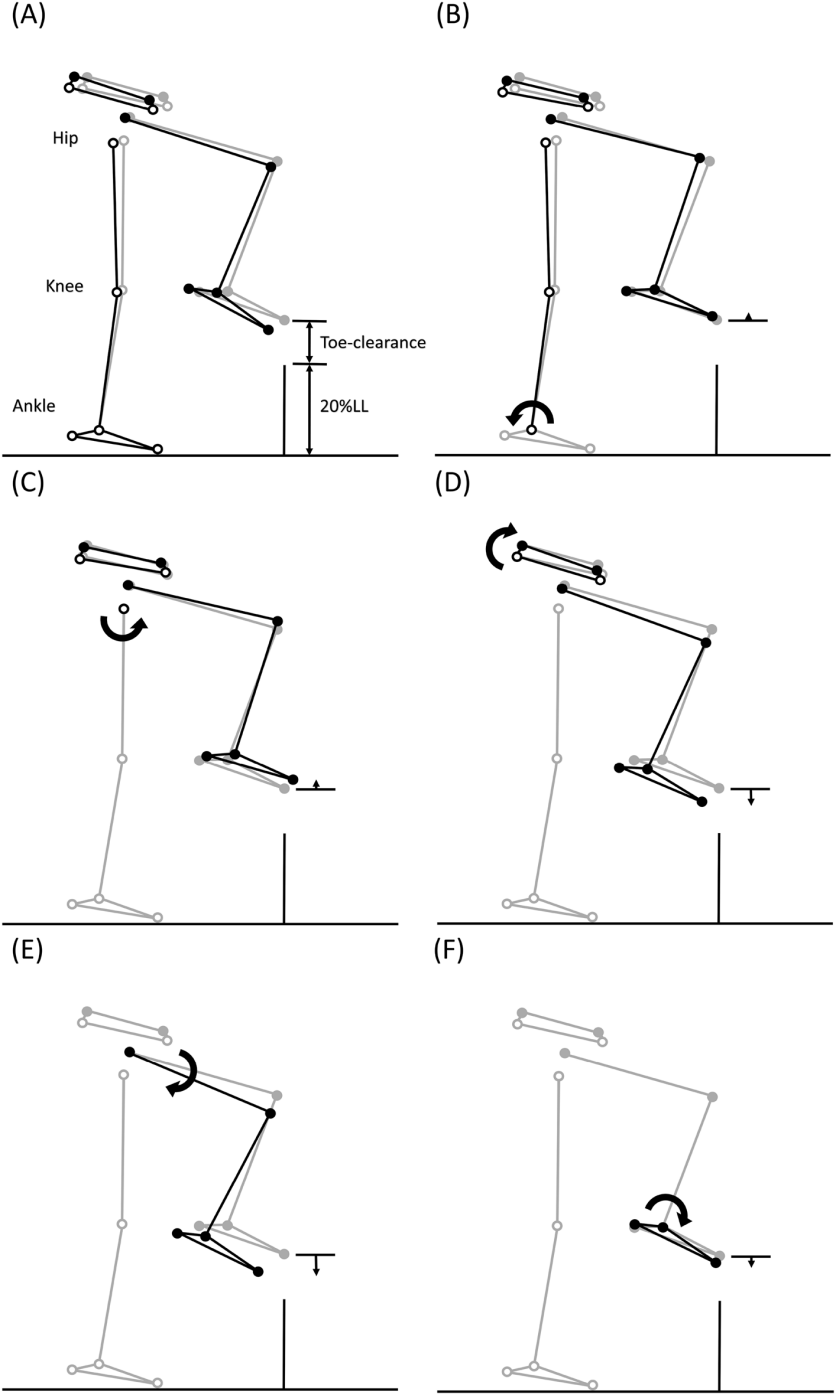
Effects of significant angular changes at individual joints on the leading toe-obstacle clearance in the sagittal plane. (**A**) Postural and end-point position differences between the DMD group (black stick figure) and the Control group (grey stick figure) when crossing an obstacle of 20% leg length. For the DMD group, while increased stance-limb ankle plantarflexion (**B**) and decreased stance-limb hip flexion (**C**) tended to increase the toe-obstacle clearance and increased pelvic anterior tilt (**D**), decreased swing-limb hip flexion (**E**) and increased swing-limb ankle plantarflexion (**F**) had the opposite effect, resulting in the observed toe-obstacle clearance similar to that of the control group. In the stick figures, line segments with open circles at the joints are farther away from the viewer, while solid circles are closer to the viewer. With the stance-foot immobilized on the ground, the sub-figures were obtained by rotating the distal part of the pelvis-leg apparatus at one joint at a time while keeping the other joints immobilized according to the significant angular changes reported in Table 3.

**Figure 5:**
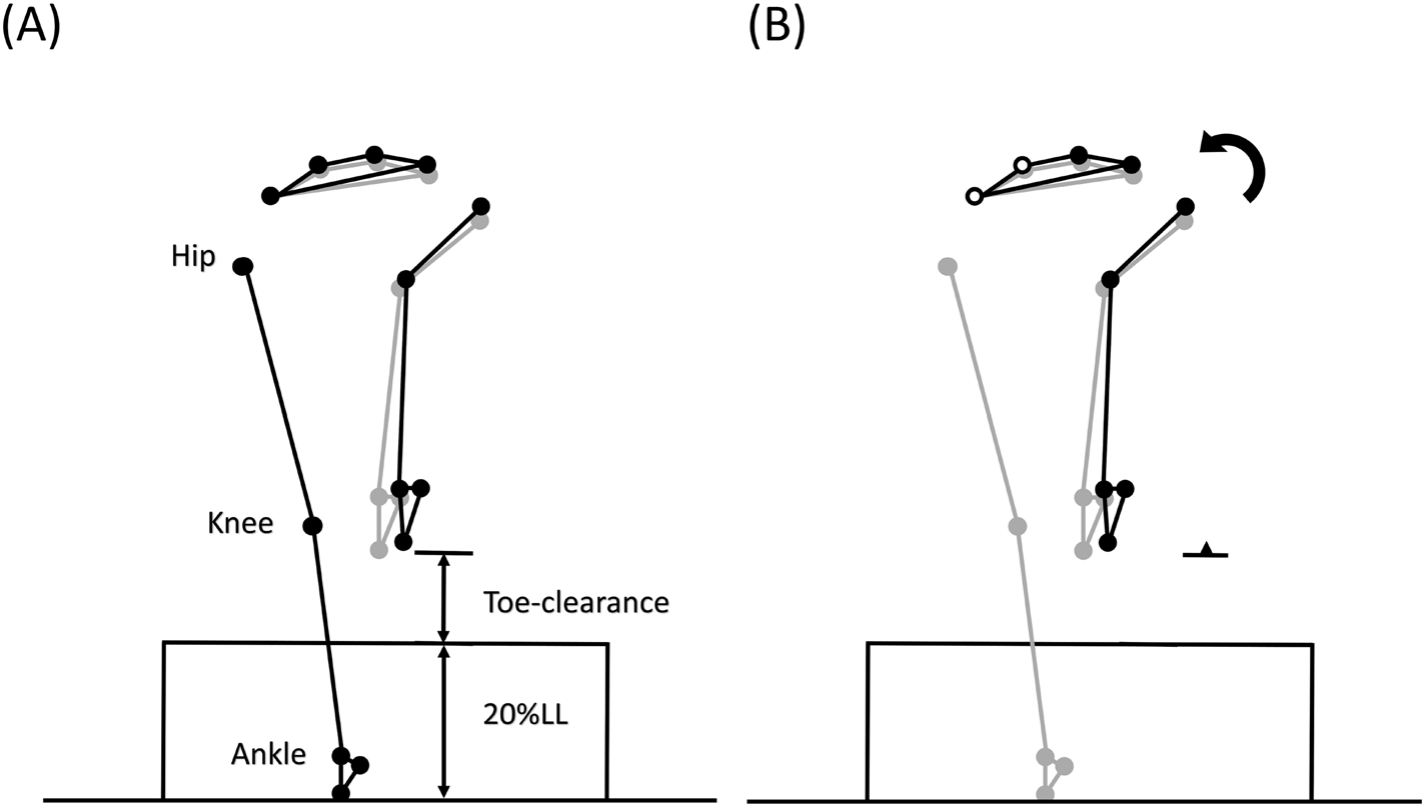
Effects of significant angular changes at individual joints on the leading toe-obstacle clearance in the frontal plane. (**A**) Postural and end-point position differences between the DMD group (black stick figure) and the Control group (grey stick figure) when crossing an obstacle of 20% leg length. For the DMD group, increased pelvic hiking (**B**) tended to increase the toe-obstacle clearance. With the stance-foot immobilized on the ground, the sub-figures were obtained by rotating the distal part of the pelvis-leg apparatus at one joint at a time while keeping the other joints immobilized according to the significant angular changes reported in Table 3.

**Figure 6:**
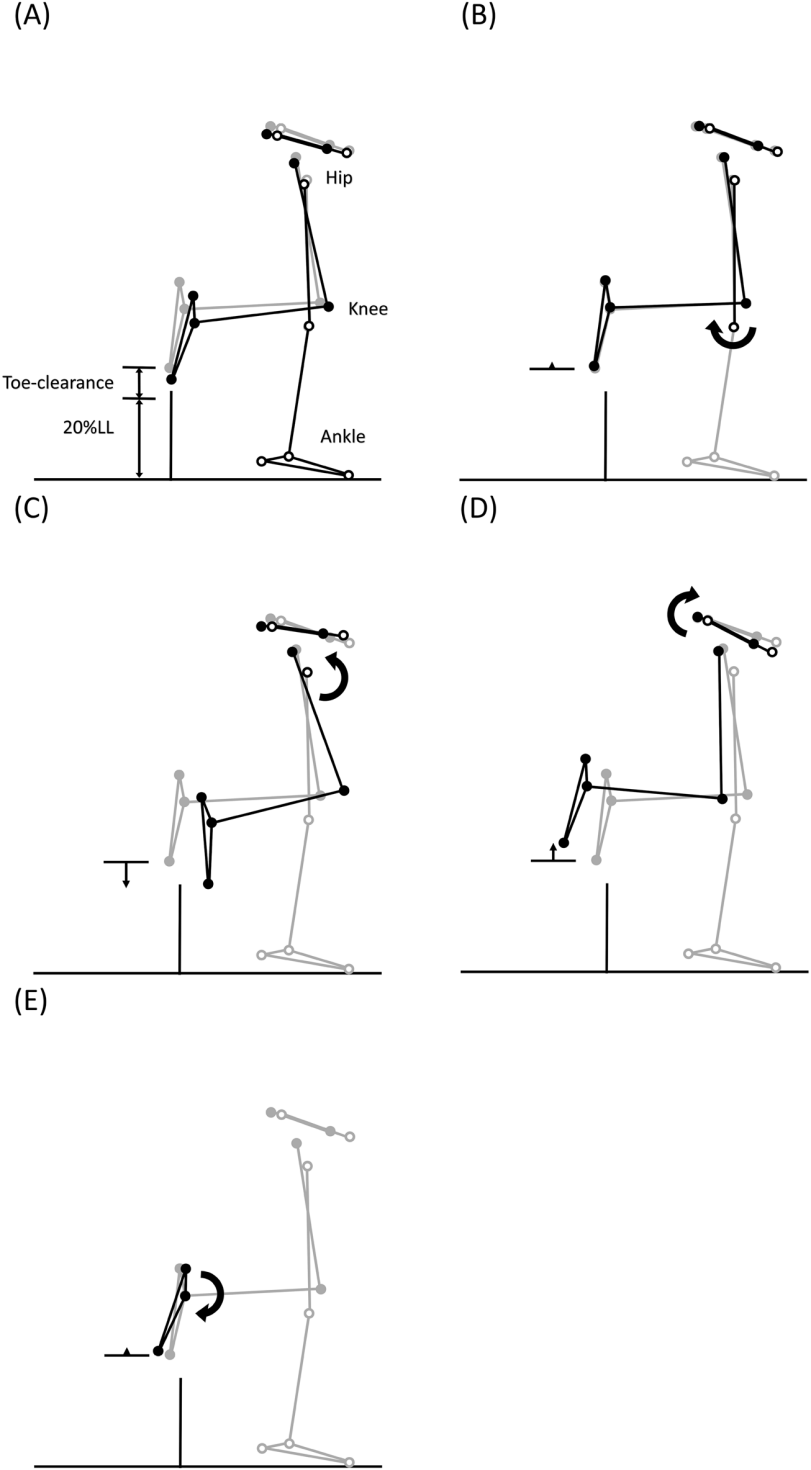
Effects of significant angular changes at individual joints on the trailing toe-obstacle clearance in the sagittal plane. (**A**) Postural and end-point position differences between the DMD group (black stick figure) and the Control group (grey stick figure) when crossing an obstacle of 20% leg length. For the DMD group, while decreased stance-limb knee flexion (**B**), increased swing-limb ankle plantarflexion (**E**) and increased pelvic anterior tilt (**D**) tended to increase the toe-obstacle clearance, decreased stance-limb hip flexion (**C**) had the opposite effect, resulting in the observed reduced toe-obstacle clearance. In the stick figures, line segments with open circles at the joints are farther away from the viewer, while solid circles are closer to the viewer. With the stance-foot immobilized on the ground, the sub-figures were obtained by rotating the distal part of the pelvis-leg apparatus at one joint at a time while keeping the other joints immobilized according to the significant angular changes reported in Table 4.

**Figure 7:**
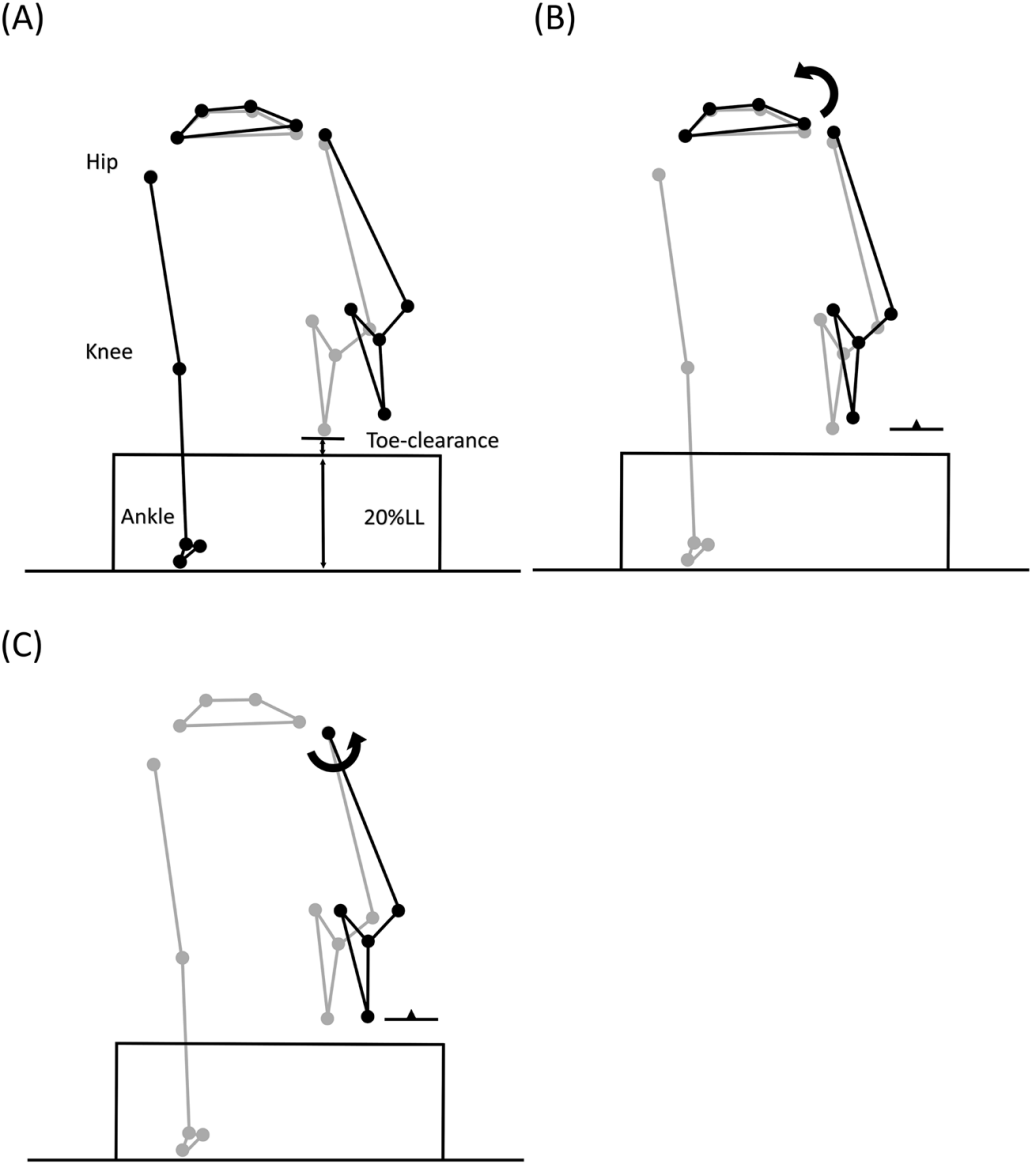
Effects of significant angular changes at individual joints on the trailing toe-obstacle clearance in the frontal plane. (**A**) Postural and end-point position differences between the DMD group (black stick figure) and the Control group (grey stick figure) when crossing an obstacle of 20% leg length. For the DMD group, both increased pelvic hiking (**B**) and hip abduction (**C**) tended to increase the toe-obstacle clearance. With the stance-foot immobilized on the ground, the sub-figures were obtained by rotating the distal part of the pelvis-leg apparatus at one joint at a time while keeping the other joints immobilized according to the significant angular changes reported in Table 4.

The normality was found in all the calculated variables. Compared to the Control, the DMD group showed significantly reduced crossing speeds, crossing step length, leading heel-obstacle horizontal distance and trailing toe-obstacle clearance, but increased crossing step width (Table 2). The two groups showed qualitatively similar patterns in the motions of the pelvis, trunk, trunk/pelvis, and lower limb joints, but quantitative differences were found in some kinematic components when either the leading toe or the trailing toe was above the obstacle (Figs. 1, 2, 3, 4, 5, 6 and 7).

**Table 2:** Means (standard deviations) of the temporal-spatial and end-point parameters in the Duchenne muscular dystrophy (DMD) and typically developed control (Control) groups when crossing obstacles of heights of 10%, 20% and 30% of the subject’s leg length (LL).

Parameters	Obstacle Height (%LL)	DMD	Control	P_G_	P_H_
Crossing speed (mm/s)	10	881.1 (134.8)	963.5 (145.1)	< 0.01*	< 0.01*
	20	741.8 (146.8)	908.0 (157.9)		
	30	702.2 (127.3)	806.2 (153.7)		
Crossing step length (mm)	10	508.5 (34.6)	566.9 (56.6)	< 0.01*	0.35
	20	530.2 (35.4)	580.5 (43.6)		
	30	527.6 (42.6)	567.5 (54.8)		
Crossing step width (mm)	10	143.4 (46.9)	95.8 (39.4)	< 0.01*	0.48
	20	153.4 (40.2)	91.7 (40.9)		
	30	154.1 (54.2)	113.6 (54.7)		
Leading toe-obstacle clearance (mm)	10	88.9 (26.9)	93.4 (47.7)	0.23	0.10
	20	100.4 (26.7)	108.6 (59.6)		
	30	75.6 (29.2)	91.4 (34.1)		
Leading heel-obstacle clearance (mm)	10	153.2 (36.9)	115.4 (37.1)	0.01*	0.49
	20	157.4 (34.0)	135.4 (65.1)		
	30	140.6 (38.5)	133.5 (38.5)		
Trailing toe-obstacle horizontal distance (mm)	10	180.51 (29.3)	174.22 (34.9)	0.299	0.730
	20	193.28 (43.9)	178.40 (37.2)		
	30	189.18 (36.3)	181.90 (61.5)		
Trailing toe-obstacle clearance (mm)	10	73.22 (42.6)	98.89 (57.8)	0.003*	0.710
	20	78.25 (51.9)	118.07 (53.4)		
	30	71.18 (51.6)	117.10 (34.8)		
Trailing heel-obstacle clearance (mm)	10	252.52 (29.7)	219.85 (135.4)	0.651	0.880
	20	251.71 (37.3)	245.41 (117.7)		
	30	233.10 (42.4)	242.32 (103.6)		
Leading heel-obstacle horizontal distance (mm)	10	132.34 (15.16)	150.66 (21.9)	< 0.002*	0.632
	20	124.93 (19.37)	140.34 (16.9)		
	30	125.83 (26.93)	145.32 (13.2)		

LL: leg length; P_G_ = group effect; P_H_ = obstacle height effect; *: P_G_ < 0.05; ↓: significant linearly decreasing trend (P_H_ < 0.05).

When the leading toe was above the obstacle, the DMD group showed significantly increased trunk and pelvic anterior tilt, trunk/pelvis extension as well as increased ipsilateral pelvic hiking and trunk/pelvis side-bending (Table 3 and Fig. 1), with significantly increased ankle plantarflexion but decreased hip flexion in both the leading and trailing limbs as compared to the Control (Table 3 and Figs. 2 and 3). These significant angular changes showed different effects on the leading and trailing toe-obstacle clearances in the DMD group, some tending to increase the toe-obstacle clearance while others showed opposite effects, leading to an unaltered leading toe-obstacle clearance (Figs. 4 and 5). When the trailing toe was above the obstacle, the DMD group showed significantly increased pelvic anterior tilt, trunk/pelvis extension and contralateral pelvic hiking but decreased contralateral trunk and trunk/pelvis side-bending (Table 4 and Fig. 1), with significantly increased hip abduction and ankle plantarflexion in the trailing limb, but decreased hip flexion and knee flexion in the leading limb (Table 4 and Figs. 2 and 3). These significant angular changes in the pelvic orientations and at individual joints affected the trailing toe-obstacle clearance in the DMD group differently, but the net effects led to a significantly reduced trailing toe-obstacle clearance (Fig. 6 and 7).

**Table 3:** Means (standard deviations) of the pelvic and trunk orientations and the crossing angles of the trunk/pelvis, hips, knees and ankles in patients with Duchenne muscular dystrophy (DMD) and typically developed controls (Control) when the leading toe was above the obstacles.

Variables (deg)	Group	Obstacle height (%LL)			*P* vValue
		10%	20%	30%	P_G_, P_H_
Pelvis relative to global					
Anterior ( + )/Posterior ( − ) Tilt	DMD	16.0 (6.6)	12.6 (7.1)	11.0 (7.2)	< 0.001*, < 0.001↓
	Control	11.2 (4.2)	8.5 (2.7)	7.4 (3.4)	
Hiking ( + )/Dropping ( − )	DMD	6.4 (2.5)	10.9 (3.5)	15.0 (3.9)	< 0.001*, < 0.001↑
	Control	4.7 (2.4)	8.1 (2.8)	10.5 (3.3)	
Trunk relative to global					
Anterior ( + )/Posterior ( − ) Tilt	DMD	6.5 (4.2)	7.5 (5.3)	8.1 (2.4)	0.003*, 0.762
	Control	3.7 (6.9)	4.8 (5.3)	2.4 (6.1)	
Ipsilateral ( + )/Contralateral ( − ) SB	DMD	0.2 (2.1)	2.7 (1.5)	4.5 (1.7)	0.198, < 0.001↑
	Control	1.4 (1.6)	3.8 (1.9)	3.7 (2.2)	
Trunk relative to pelvis					
Flexion ( + )/Extension ( − )	DMD	− 7.7 (3.4)	− 6.1 (4.6)	− 3.1 (4.2)	0.011*, < 0.001↑
	Control	− 5.8 (5.9)	− 3.3 (4.4)	− 2.7 (3.5	
Ipsilateral ( + )/Contralateral ( − ) SB	DMD	6.2 (1.1)	8.6 (2.3)	9.7 (3.8)	< 0.001*, < 0.001↑
	Control	3.6 (0.9)	4.6 (1.3)	6.5 (3.4)	
Leading swing− limb					
Hip					
Flexion ( + )/Extension ( − )	DMD	49.7 (8.8)	60.8 (12.9)	65.9 (10.3)	0.036*, < 0.001↑
	Control	51.7 (9.0)	68.0 (8.6)	72.4 (9.4)	
Abduction ( + )/Adduction ( − )	DMD	− 9.3 (5.8)	− 17.2 (21.4)	− 28.5 (15.1)	0.117, < 0.001↓
	Control	− 3.3 (7.0)	− 9.0 (12.6)	− 26.3 (12.9)	
Knee					
Flexion ( + )/Extension ( − )	DMD	81.5 (8.0)	92.1 (9.0)	98.3 (9.3)	0.946, < 0.001↑
	Control	77.3 (10.9)	95.4 (5.4)	99.6 (4.3)	
Abduction ( + )/Adduction ( − )	DMD	− 1.8 (2.3)	− 2.2 (2.1)	1.7 (1.8)	0.959, 0.487
	Control	− 0.1 (2.2)	0.1 (1.9)	− 0.6 (1.7)	
Ankle					
Dorsiflexion ( + )/Plantarflexion ( − )	DMD	− 5.9 (4.6)	− 5.2 (5.8)	− 4.5 (4.9)	< 0.001*, 0.419
	Control	2.2 (5.6)	2.4 (6.2)	4.9 (4.4)	
Abduction ( + )/Adduction ( − )	DMD	− 3.2 (4.9)	− 3.5 (5.2)	− 1.5 (4.4)	0.231, 0.072
	Control	− 0.3 (2.9)	− 4.7 (4.4)	− 1.3 (3.5)	
Trailing stance− limb					
Hip					
Flexion ( + )/Extension ( − )	DMD	− 2.8 (2.6)	− 3.9 (2.4)	− 4.5 (4.6)	0.018*, 0.291
	Control	− 0.7 (3.1)	− 1.2 (2.9)	− 2.4 (4.9)	
Abduction ( + )/Adduction ( − )	DMD	3.6 (3.8)	3.4 (5.3)	4.3 (5.4)	0.286, 0.309
	Control	2.7 (2.0)	4.3 (3.1)	5.9 (2.6)	
Knee					
Flexion ( + )/Extension ( − )	DMD	3.9 (3.7)	5.0 (3.7)	6.0 (5.9)	0.288, 0.224
	Control	5.0 (1.9)	6.0 (3.3)	7.3 (5.5)	
Abduction ( + )/Adduction ( − )	DMD	− 2.3 (4.3)	− 2.9 (2.5)	− 3.9 (5.3)	0.312, 0.640
	Control	− 3.2 (3.2)	− 4.1 (3.3)	− 6.4 (2.3)	
Ankle					
Dorsiflexion ( + )/Plantarflexion ( − )	DMD	− 2.0 (2.6)	− 3.4 (3.2)	− 4.8 (3.9)	0.043*, 0.040↓
	Control	− 0.3 (2.7)	− 1.3 (2.9)	− 2.8 (4.8)	
Abduction ( + )/Adduction ( − )	DMD	− 2.1 (3.6)	− 1.9 (5.6)	− 1.4 (6.1)	0.085, 0.143
	Control	− 1.5 (3.4)	− 2.4 (3.2)	− 2.2 (4.2)	

SB: side-bending; LL: leg length; P_G_ = group effect; P_H_ = obstacle height effect; *: P_G_ < 0.05; ↑: significant linearly increasing trend; ↓: significant linearly decreasing trend (P_H_ < 0.05).

**Table 4:** Means (standard deviations) of the pelvic and trunk orientations and the crossing angles of the trunk/pelvis, hips, knees and ankles in patients with Duchenne muscular dystrophy (DMD) and typically developed controls (Control) when the trailing toe was above the obstacles.

Variables (deg)	Group	Obstacle height (%LL)			*P* value
		10%	20%	30%	P_G_, P_H_
Pelvis relative to global					
Anterior ( + )/Posterior ( − ) Tilt	DMD	22.6 (7.0)	23.1 (7.5)	24.8 (7.7)	< 0.001*, 0.737
	Control	14.5 (4.9)	14.8 (6.9)	15.6 (8.7)	
Hiking ( + )/Dropping ( − )	DMD	− 1.7 (3.7)	− 5.5 (8.0)	− 10.5 (7.6)	0.049*, < 0.001↓
	Control	− 0.1 (3.7)	− 1.7 (2.5)	− 7.9 (3.9)	
Trunk relative to global					
Anterior ( + )/Posterior ( − ) Tilt	DMD	8.5 (5.6)	4.9 (3.2)	6.1 (4.4)	0.192, 0.894
	Control	3.8 (4.5)	6.4 (2.8)	5.1 (4.2)	
Ipsilateral ( + )/Contralateral ( − ) SB	DMD	0.2 (4.2)	5.2 (4.5)	12.7 (5.3)	< 0.001*, < 0.001↑
	Control	− 2.8 (4.0)	− 1.4 (1.8)	7.6 (4.3)	
Trunk relative to pelvis					
Flexion ( + )/Extension ( − )	DMD	− 18.8 (5.)	− 15.9 (4.5)	− 17.2 (4.4)	< 0.001*, 0.463
	Control	− 11.3 (4.5)	− 12.4 (4.5)	− 10.0 (3.9)	
Ipsilateral ( + )/Contralateral ( − ) SB	DMD	− 1.8 (1.8)	− 0.5 (2.3)	2.3 (1.6)	< 0.001*, < 0.001↑
	Control	− 3.0 (1.8)	− 4.0 (1.7)	0.6 (1.5)	
Trailing swing− limb					
Hip					
Flexion ( + )/Extension ( − )	DMD	9.1 (9.0)	15.2 (11.3)	23.0 (21.0)	0.867, 0.025↑
	Control	12.0 (8.1)	17.7 (6.4)	17.2 (5.7)	
Abduction ( + )/Adduction ( − )	DMD	− 5.7 (6.5)	− 13.6 (10.5)	− 21.9 (13.5)	< 0.001*, < 0.001↓
	Control	− 3.1 (4.0)	− 5.2 (6.6)	− 7.8 (6.9)	
Knee					
Flexion ( + )/Extension ( − )	DMD	82.5 (10.4)	93.6 (10.8)	99.4 (11.6)	0.350, < 0.001↑
	Control	81.8 (11.6)	97.0 (9.0)	103.9 (8.7)	
Abduction ( + )/Adduction ( − )	DMD	− 5.0 (3.2)	− 3.4 (1.4)	− 0.7 (4.2)	0.523, 0.091
	Control	− 3.5 (2.7)	− 0.4 (3.2)	− 1.2 (5.9)	
Ankle					
Dorsiflexion ( + )/Plantarflexion ( − )	DMD	− 21.3 (5.7)	− 19.4 (6.9)	− 16.3 (6.3)	< 0.001*, 0.040↑
	Control	− 11.5 (11.1)	− 7.3 (10.8)	− 2.5 (10.8)	
Abduction ( + )/Adduction ( − )	DMD	− 2.3 (2.6)	− 3.9 (3.5)	− 1.7 (4.1)	0.524, 0.633
	Control	− 2.4 (6.2)	− 3.1 (4.2)	− 2.1 (6.7)	
Leading stance− limb					
Hip					
Flexion ( + )/Extension ( − )	DMD	− 3.0 (10.7)	− 1.3 (10.3)	− 1.6 (11.1)	< 0.001*, 0.812
	Control	7.9 (7.8)	10.1 (10.7)	8.5 (9.0)	
Abduction ( + )/Adduction ( − )	DMD	− 0.7 (4.1)	− 0.3 (4.5)	− 0.7 (4.9)	0.697, 0.461
	Control	0.6 (3.0)	0.4 (2.7)	− 1.7 (1.9)	
Knee					
Flexion ( + )/Extension ( − )	DMD	7.8 (7.5)	8.3 (7.5)	6.5 (5.6)	0.004*, 0.506
	Control	10.7 (4.8)	9.7 (5.9)	8.1 (4.7)	
Abduction ( + )/Adduction ( − )	DMD	− 2.5 (2.7)	− 3.2 (6.2)	− 2.2 (3.4)	0.078, 0.089
	Control	− 2.5 (3.3)	− 4.2 (5.2)	− 2.5 (7.7)	
Ankle					
Dorsiflexion ( + )/Plantarflexion ( − )	DMD	− 4.3 (3.0)	− 5.9 (2.4)	− 6.0 (2.5)	0.083, 0.024↓
	Control	− 1.5 (2.8)	− 3.7 (3.0)	− 6.1 (6.6)	
Abduction ( + )/Adduction ( − )	DMD	− 2.3 (2.6)	− 3.1 (2.6)	− 2.8 (4.0)	0.560, 0.328
	Control	− 0.3 (6.0)	− 0.9 (7.2)	− 0.3 (7.1)	

SB: side-bending; LL: leg length; P_G_ = group effect; P_H_ = obstacle height effect; *: P_G_ < 0.05; ↑: significant linearly increasing trend; ↓: significant linearly decreasing trend (P_H_ < 0.05).

There were no interactions for the two-way mixed-design ANOVA for any of the calculated variables. With increasing obstacle height, both DMD and Control groups reduced their crossing speeds linearly (Table 2). When the leading toe was above the obstacle, both groups linearly increased the pelvic hiking and ipsilateral trunk and trunk/pelvis side-bending, as well as increased swing-limb knee flexion, and hip flexion and abduction, but linearly decreased pelvic anterior tilt and trunk/pelvis extension, as well as linearly increased ankle plantarflexion of the stance-limb (Table 3). On the other hand, when the trailing toe was above the obstacle, both groups linearly increased contralateral pelvic hiking and ipsilateral trunk side-bending, swing-limb hip flexion and abduction, swing-limb knee flexion, and stance-limb ankle plantarflexion, but linearly decreased contralateral trunk/pelvis side-bending and swing-limb ankle plantarflexion (Table 4).

## Discussion

The current study aimed to identify the kinematic strategies of the trunk-pelvis-leg apparatus in children with DMD when crossing obstacles of three different heights. During leading-limb crossing, children with DMD showed a toe-obstacle clearance similar to that of their typically developed peers but with altered pelvis-leg kinematics, namely increased pelvic anterior tilt and hiking, and increased ankle plantarflexion, but decreased hip flexion in both limbs. Such kinematic features were not affected by obstacle heights of up to 30% of the leg length, as indicated by the independence between the height and group effects (Tables 2, 3, 4 and Figs. 2 and 3). Increases in the pelvic anterior tilt and ankle plantarflexion angular changes were also found during level walking in patients with DMD^17^. Increased pelvic hiking and the changes in the stance-ankle and -hip tended to increase the leading toe-obstacle clearance, while the increased pelvic anterior tilt and changes in the swing-ankle and hip tended to do the opposite (Figs. 3 and 4). It appears that the potentially unfavorable downward deviations of the swing-toe owing to the increased anterior pelvic tilt and changes in the swing-limb were compensated for by the effects of the kinematic changes in the trailing stance-limb.

During trailing-limb crossing, however, the kinematic compensation strategy adopted was not successful in maintaining a normal toe-obstacle clearance. Instead, the children with DMD showed a significantly reduced toe-obstacle clearance. They showed decreased hip flexion of the leading stance-limb, which tended to decrease the trailing toe-obstacle clearance (Fig. 5). However, the significantly increased pelvic anterior tilt and hiking, hip abduction and ankle plantarflexion of the trailing swing-limb did not produce enough upward deviation of the swing-toe to compensate for the decrease owing to the decreased hip flexion of the stance-limb (Figs. 5 and 6). Note that hip abduction and ankle plantarflexion of the trailing swing-limb mainly deviated the swing-toe horizontally without much influence on the toe-obstacle clearance. The current results showed that the observed toe-obstacle clearance, which has been shown to be related to the risk of tripping^46^, was mainly determined by changes in the sagittal plane kinematics. While the frontal plane kinematic changes had less influence on the toe-obstacle clearance, they have been shown to play an essential role in the frontal plane balance control during obstacle-crossing^47–49^.

Apart from the kinematic changes of the pelvis-leg apparatus, those of the trunk also played an important part in the specific kinematic strategies for obstacle-crossing observed in the patients with DMD. Being the largest body segment, the trunk represents more than half of the body mass, so its angular changes affect the kinematic interactions of the body segments^35,50–52^. During leading limb crossing, increased anterior tilt of the trunk and pelvis may help the forward progression of the body and the reduction of the loads to the leading stance limb during weight acceptance after crossing while maintaining whole body balance^53–55^. On the other hand, during the trailing crossing, the DMD group decreased significantly the contralateral trunk side-bending, indicating a shift of the body weight towards the leading stance limb to reduce the demands of the stance-hip abductor muscles similar to the Trendelenburg’s sign^56–58^. A similar trunk kinematic pattern was also found in people with poor lower-limb strength, which may have an increased risk of falling when tripping or losing balance, owing to the anterior and lateral inclination of their posture^54,59^. Further study is needed to examine the effects of the observed trunk-pelvis-leg kinematic changes on the stability of the stance limb and whole-body balance during obstacle-crossing in the DMD population.

As a genetic disease characterized by progressive muscle weakness from the proximal lower limbs to the distal, the observed changes in the end-point and trunk-pelvis-leg kinematics in the DMD group can be attributed to weakness in specific muscles (Table 1)^5,60,61^. From a neuromechanical viewpoint, increased pelvic anterior tilt and trunk/pelvis extension may be associated with the weakness of the gluteus maximus, while decreased hip flexion may be due to the weakness of the rectus femoris (Table 1). Moreover, reduced strength of the tibialis anterior muscle may be related to the increased ankle plantarflexion during obstacle-crossing (Tables 1, 3 and 4). Altered side-bending of the trunk during trailing crossing was likely an effective compensation mechanism for the weakness of the hip abductor and lower limb muscles in patients with DMD, a strategy that has been reported in various locomotor activities in the DMD population^12,62–65^. Further studies with simultaneous kinematic and electromyography measurements would be helpful for a complete understanding of the motor control strategies in children with DMD during obstacle-crossing.

Significantly reduced trailing toe-obstacle clearance indicates an increased risk of tripping in children with DMD, especially because neither the swing-limb nor the obstacle is within the subject’s visual field during trailing-limb crossing^32^. Vision integrates predicting the relative positions of the body segment and the obstacle during crossing, and visual feedback significantly influences the end-point control strategy during obstacle-crossing^66^, without which the risk of tripping would be increased^67^. Children with DMD were found to rely on visual feedback for motor control because they showed compromised manipulation abilities, especially in the absence of visual feedback^66^. Therefore, crossing obstacles with the trailing limb presents a greater challenge in sensorimotor control than crossing with the leading limb for children with DMD.

The current study was the first attempt in the literature to identify the kinematic changes of the trunk and the individual joints of the pelvis-leg apparatus, and their relationship with the end-point positions, revealing the kinematic strategies used by children with DMD during obstacle-crossing. The syntheses of the relationship were limited to the sagittal and frontal planes. The transverse rotations and thus effects on the end-point positions were expected to be small, but further studies may help confirm the current findings. Further study on the joint kinetics, body's center-of-mass motion, and muscle activities may be needed for a better insight into how the neuromusculoskeletal system is controlled to compensate for weakness of specific muscles, and the effects of this strategy on balance control during obstacle-crossing in patient with DMD. Further study using the current approach may be also needed to identify the risk factors for falling during obstacle-crossing for patients at different stages of DMD.

## Conclusions

The current study identified the kinematic strategies of the trunk-pelvis-leg apparatus for obstacle-crossing specific to children with DMD as compared to typically developed peers. The observed kinematic strategy, which was related to the weakness of specific muscles, successfully maintained a normal toe-obstacle clearance during leading-limb crossing but failed to reach that necessary for a normal trailing-limb crossing. The significantly reduced trailing toe-obstacle clearance indicates an increased risk of tripping in children with DMD, especially because neither the swing-limb nor the obstacle is within the subject’s visual field during trailing-limb crossing. The observed toe-obstacle clearance was found to be determined mainly by the sagittal plane kinematic changes. Thus, regular monitoring of such kinematic components during obstacle-crossing in children with DMD is necessary for early detection of any signs of inability to execute the observed strategies or for reduced foot-obstacle clearance leading to an increased risk of falling. The current results provide baseline data which will be helpful for the design of assistive devices or development of rehabilitation programs for tripping risk management during obstacle-crossing in patients with DMD.

## Data Availability

The datasets used and/or analysed during the current study available from the corresponding author on reasonable request.
